# Larvicidal Activity of Extracts from the *Artemisia arborescens* L. Plant and *Hyrtios erectus* Sponge Against the *Culex pipiens* Mosquito (Diptera: Culicidae) and Toxicological Assessment on *Danio rerio* Zebrafish Embryos as Non-Target Organism

**DOI:** 10.3390/insects16050448

**Published:** 2025-04-24

**Authors:** Sadeem A. Alqurashi, Ashraf M. Ahmed, Ali A. El Gamal, Shaza M. Al-Massarani, Omer A. Basudan, Diaa T. A. Youssef, Lamiaa A. Shaala, Muhammad Farooq Khan

**Affiliations:** 1 Department of Zoology, College of Science, King Saud University, Riyadh 11451, Saudi Arabia; 443204430@student.ksu.edu.sa (S.A.A.); aalii@ksu.edu.sa (A.M.A.); 2 Department of Pharmacognosy, College of Pharmacy, King Saud University, P.O. Box 2457, Riyadh 11451, Saudi Arabia; 3 Department of Natural Products, Faculty of Pharmacy, King Abdulaziz University, Jeddah 21589, Saudi Arabia; 4 Suez Canal University Hospitals, Suez Canal University, Ismailia 41522, Egypt

**Keywords:** bioinsecticides, *Danio rerio*, ecofriendly, embryotoxicity, mosquitocidal, Red Sea sponges, vector control

## Abstract

There is a growing need for eco-friendly methods to control disease-carrying insects. The present study aimed to investigate the larvicidal activity of methanolic extracts and their various fractions from a plant, *Artemisia arborescens* L., and a marine sponge, *Hyrtios erectus*, against the mosquito, *Culex pipiens* L. Crude methanolic extracts of *A. arborescens* and *H. erectus* were prepared by maceration and successive fractionation were obtained using the liquid–liquid partition of crude extracts. The larvicidal activity of the extracts and their fractions was determined according to the WHO standard method. The results revealed that the *n*-hexane fraction of *A. arborescens* exhibited the highest larvicidal activity (LC_50_ 346.74 μg/mL), exceeding the efficacy of the crude extract and other fractions. Furthermore, the sponge’s *n*-hexane (LC_50_ 68.39 μg/mL), chloroform (LC_50_ 63.03 μg/mL), and *n*-butanol (LC_50_ 71.23 μg/mL) fractions showed a significant 3.9 to 4.5 times increase in the larvicidal potency compared to its crude extract (LC_50_ = 280.74 μg/mL). The safety of the sponge extracts was tested in the embryos of zebrafish as a non-target organism. In this regard, the crude methanolic extract and *n*-butanol fraction exhibited weak toxicity and chloroform fraction showed no detectable toxicity. This study suggests the *H. erectus* sponge as a source for developing safe natural substitutes for use in the battle against *Cx. pipiens* mosquito, which may help in reducing the spread of mosquito vectors and mosquito-borne diseases.

## Simple Summary

The study investigates the larvicidal activity of methanolic extracts from *Artemisia arborescens* and *Hyrtios erectus* sponge against *Culex pipiens* mosquitoes. The extracts were prepared via maceration and were further fractionated using solvents of variable polarity. The results showed a significant difference in activity; the *n*-hexane fraction of *A. arborescens* had the highest effect, while *H. erectus* demonstrated greater overall larvicidal potency. Safety assessments revealed low toxicity of sponge extract on zebrafish embryos, suggesting *H. erectus* as a promising natural alternative for controlling mosquito populations and related diseases.

## 1. Introduction

Mosquitoes constitute a significant health threat worldwide, transmitting a wide range of deadly diseases that affect millions of people every year [1]. The *Culex pipiens* mosquito, commonly known as the house mosquito [2], holds significant medical importance due to its ability to transmit various human and animal pathogens [3]. This mosquito is a critical vector of arboviruses such as the West Nile virus (WNV) [4,5], St. Louis encephalitis virus (SLEV) [6], and the Usutu virus [7], which cause severe neurological disorders in humans. It also transmits the parasitic nematode responsible for lymphatic filariasis in humans [8], as well as avian malaria parasites (*Plasmodium* spp.), which threaten bird populations [9]. Its ability to adapt to urban and suburban environments frequently brings it into close contact with human populations, making it a significant global public health concern and necessitating continuous efforts to control its spread and reduce disease transmission [3,10]. Conventional synthetic pesticides, while still crucial in mosquito control, have significant drawbacks. Widespread use has resulted in adverse environmental impacts, including harm to non-target organisms, the emergence of pesticide-resistant mosquitoes, disruption of beneficial insects, outbreaks of secondary pests, and the accumulation of harmful residues in the environment [11,12,13]. These challenges, coupled with limited vaccines and treatments for many mosquito-borne diseases [14], have driven a growing demand within the research community to develop more sustainable and eco-friendly mosquito control strategies from natural sources.

Plant-derived insecticides have gained attention as sustainable alternatives to synthetic chemicals due to their biodegradability, low environmental persistence, and reduced risk of resistance development [15,16,17]. Among the diverse array of plants with insecticidal properties, the *Artemisia* genus, belonging to the Asteraceae family, stands out due to its rich repertoire of bioactive compounds. These plants produce a wide spectrum of secondary metabolites, including monoterpenes, sesquiterpenes, flavonoids, and phenolic compounds, all contributing to their potent insecticidal activity. The synergistic interactions between these compounds amplify their effectiveness, making members of the genus *Artemisia* particularly potent [18]. *Artemisia* extracts have demonstrated efficacy in controlling a broad range of insect pests in agriculture, stored-product protection, and public health, showcasing their versatility and potential [19].

On the other hand, the marine environment stands as an exceptional source of bioactive natural products, teeming with a diverse array of novel structures boasting unique biological properties often unparalleled in terrestrial sources. This remarkable richness can be attributed to the vast biodiversity and extreme conditions characteristic of marine ecosystems [20]. Marine sponges (Phylum: Porifera), the most ancient metazoan organisms, have emerged as a rich reservoir of biologically active compounds with significant potential for human applications. These organisms are increasingly recognized as promising alternatives to synthetic pesticides [21] and a valuable source of novel antimicrobial agents [22]. The diversity of marine sponges is extensive, with approximately 9432 species described globally [23]. However, only very few of these species have been investigated for their mosquito larvicidal properties, indicating a largely untapped potential in this field [24].

The current study aims to assess and compare the larvicidal activity of crude methanolic extracts and their fractions from the *Artemisia arborescens* (Vaill.) L. plant and the marine sponge *Hyrtios erectus* (Keller, 1889) against *Culex pipiens*, Linnaeus, 1758. Subsequently, the extracts showing the highest potency against mosquito larvae underwent safety testing via the zebrafish, *Danio rerio* (Hamilton, 1822), embryonic toxicity assay as a non-target organism.

## 2. Materials and Methods

### 2.1. Collection of Plant Materials

Aerial parts (leaves, stems, and flowers) of the plant *A. arborescens* (Tracheophyta: Asterales: Asteraceae) were harvested from the Jazan region, Saudi Arabia, during the flowering stage. The plant material was thoroughly rinsed with tap water to remove dust and surface contaminants, subsequently shade-dried at ambient temperature, and ground into a fine powder. Taxonomic identification was performed by Dr. Mohammed Yusuf, taxonomist of the Medicinal, Aromatic, and Poisonous Plants Research Center (MAPPRC), College of Pharmacy, King Saud University, Riyadh, Saudi Arabia. A voucher specimen (#16079) was archived in the Pharmacognosy Department’s repository at the same institution.

### 2.2. Collection of a Marine Sponge

**Figure 1:**
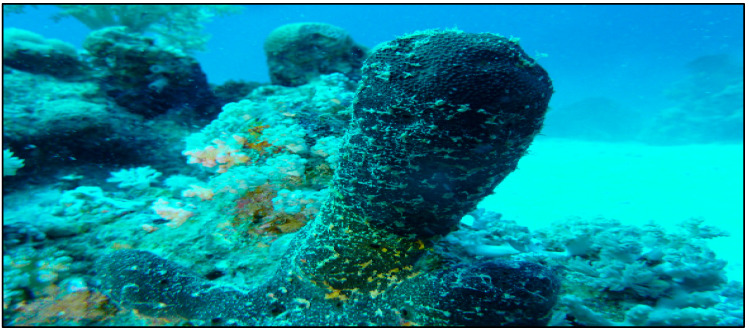
Underwater (−30 m) photograph of the Red Sea sponge *H. erectus*.

The *H. erectus* (Figure 1) marine sponge (Porifera: Dictyoceratida: Thorectidae) was collected by SCUBA diving at a depth of −30 m off Yanbu at the Saudi Red Sea Coast. The sponge specimens were gently detached by hand from the substrate, placed in plastic bags underwater, and frozen directly after collection. After transferring to the laboratory, the sponge materials were freeze-dried to ensure an efficient extraction process. The collected specimen was kindly identified by Rob van Soest. A sponge specimen was deposited at the Naturalis Museum, the Netherlands, under catalog number RMNH Por. 9178. Another specimen was deposited at the Red Sea Invertebrates Collection at King Abdulaziz University under code # DY-KSA-24.

### 2.3. Preparation of Crude Extract

Crude methanolic extracts of *A. arborescens* and *H. erectus* were prepared via room-temperature maceration according to [25]. For *A. arborescens*, 550 g of the air-dried powdered material underwent four extractions, each with 1 L of 95% methanol. The resulting extracts were filtered (Whatman No. 1) and concentrated by rotary evaporation at 40 °C under reduced pressure, yielding 13 g of crude methanolic extract. Similarly, 1.85 kg of freeze-dried *H. erectus* sponge was extracted five times, each with 2.5 L of absolute methanol. Following filtration and rotary evaporation at 40 °C, 100 g of crude extract was obtained.

#### 2.3.1. Fractionation of Crude Extract

The crude methanolic extracts of *A. arborescens* and *H. erectus* were successively partitioned to obtain the corresponding fractions by liquid–liquid extraction following the method described by Otsuka [26]. Initially, each crude extract was dissolved in a methanol/water mixture. These solutions were then partitioned against solvents of increasing polarity: *n*-hexane, dichloromethane, and ethyl acetate for plant *A. arborescens* and *n*-hexane, chloroform, and *n*-butanol for sponge *H. erectus*. Using a separating funnel, each solvent fraction was separated and collected individually. The fractions were subsequently concentrated under reduced pressure using a rotary evaporator. The concentrated fractions were further dried to ensure the complete removal of the residual solvent. The yield of each fraction was determined by weighing, and the fractions were stored in the refrigerator at 4 °C for subsequent use.

#### 2.3.2. Preparation of Stock Solution

A standard stock solution of 10 mg/mL of each crude methanolic extract and its fractions of plant and marine sponge were individually prepared by dissolving 100 mg of the dry material in 300 µL of dimethylsulfoxide (DMSO), followed by dilution with distilled water to reach a total volume of 10 mL. To ensure the complete solubility of the materials in the water, Triton X-100 (The Hartz Mountain Corporation, Secaucus, NJ, USA) was added as an emulsifier at a concentration of 0.005% in the final solution. The stock solutions were stored in a refrigerator at 4 °C to maintain stability and efficacy for subsequent experimental use.

### 2.4. Maintenance of Mosquitoes’ Colony

The house mosquito, *Cx. pipiens*, used in this study, was an insecticide-naïve laboratory strain reared in the insectary of the Zoology Department, College of Science, King Saud University for many years, and used for several similar studies in our group [27,28,29].

The information related to the strains and source of and how the mosquito colony was reared was essentially the same as described previously [30] under standardized conditions, at 28 °C and 70–80% relative humidity with a photoperiod of 12:12 (L:D) hours. Adult mosquitoes were maintained in netted cages (30 × 30 × 30 cm) and provided with a 10% sugar solution (*w*/*v*) ad libitum as an energy source. Larvae were reared in plastic trays containing dechlorinated water and fed a diet of ground Goldfish Flake Food (Warldley^®^, Las Vegas, NV, USA) until pupation. Pupae were collected daily and transferred to rearing cages for adult emergence. On the fifth to seventh day post-adult emergence, female mosquitoes were starved for 12 h before being offered a blood meal from a live, constrained pigeon for egg production. After three days, 300 mL plastic containers filled with tap water were provided to the gravid mosquitoes for oviposition. Egg rafts were collected and transferred to larval-rearing trays (30 × 20 × 12 cm).

### 2.5. Mosquito Larvicidal Bioassay

The larvicidal activity of each crude methanolic extract and its different fractions of both plant and marine sponge against the third larval instar of *Cx. pipiens* mosquitoes were evaluated according to the standard World Health Organization [31] method, with some modifications as described by Ahmed et al. [30]. The bioassays were conducted in 12-well tissue culture plates, with each well containing 10 larvae and five replicates per concentration. For *A. arborescens,* the tested concentrations included 1200, 1500, 1700, and 2000 μg/mL for the methanolic extract; 200, 300, 400, and 500 μg/mL for the *n*-hexane fraction; 1200, 1500, 1700, and 2000 μg/mL for the dichloromethane fraction; and 1800, 2000, 2300, and 2500 μg/mL for the ethyl acetate fraction. In the case of *H. erectus*, the methanolic extract was tested at 200, 300, 400, and 500 μg/mL; the *n*-hexane fraction at 60, 70, 80, and 100 μg/mL; the chloroform fraction at 50, 70, 90, and 100 μg/mL; and the *n*-butanol fraction at 60, 80, 120, and 150 μg/mL. The selection of these concentrations was determined based on preliminary screening tests. Two sets of control groups were used in the experiment, one exposed to DMSO (control-1) and the other to Triton X-100 (control-2). The bioassays lasted for 48 h, with larval mortality being observed at 24 and 48 h post-treatment.

If mortality percentages in the control groups fell within the range of 5% to 20%, Abbott’s formula [32] was used to adjust the percentage mortality in the treatments via utilizing the formula shown below:

$$Corrected\;mortality\;(\%)=\frac{\%\;treatment\;mortality-\%\;control\;mortality}{100-\%\;control\;mortality}\times 100$$

### 2.6. Evalution of Biotoxicity Against Non-Target Organisms

The extracts showing the highest larvicidal activity against mosquito larvae underwent toxicological testing. The wild-type strain of zebrafish was reared and maintained at the Bioproducts Research Chair, Department of Zoology, King Saud University, at 28 °C and photoperiod of 14 light:10 dark. The embryotoxicity assessment of the zebrafish was conducted following the method described by Abutaha et al. [33] with a slight modification. Zebrafish embryos were exposed to various concentrations of crude methanolic extract of marine sponge and its derived fractions in a 12-well plate, with a DMSO and Triton X-100 mixture serving as the control. Each well consisted of three embryos and the experiment was performed in triplicate. The plates were maintained at a constant temperature of 28 °C. Observations on embryo mortality (defined by coagulation and the absence of a heartbeat), hatchability, and malformations were made at 24, 48, and 72 h post-treatment using a dissection microscope. Images were captured using an Olympus SZ10 Stereo microscope fitted with a DP72 camera (Tokyo, Japan) and analyzed by the CellsSense standard (Olympus) software (version 3.2). The biosafety index (BI) is determined using the formula established by Moungthipmalai et al. [34] to evaluate the potential toxicity associated with the marine sponge extracts under investigation.

$$Biosafety\;index\;(BI)=\frac{{LC}_{50}\;of\;non\text{-}target\;organism}{{LC}_{50}\;of\;target\;organism}$$

A biosafety index (BI) greater than 1 indicates that the treatment is safe for non-target organisms. In contrast, a BI of less than 1 suggests that the treatment is harmful to non-target organisms. The study was approved by the Research Ethics Committee at King Saud University, Riyadh, Saudi Arabia, with the approval reference number KSU-SE-23-61 dated 21 June 2023.

### 2.7. Statistical Analysis

The larvicidal bioassay data underwent Probit analysis following Finney’s method [35] to determine the LC_50_ and LC_90_ values (µg/mL) along with their 95% confidence limits (lower and upper limits), Slope ± SE, and Chi-square value utilizing the LdP Line software available online: https://www.ehabsoft.com/ldpline/ (accessed on 24 February 2025). Treatments were deemed non-significantly different in toxicity if the 95% confidence intervals overlapped, as observed by [36].

## 3. Results

### 3.1. Mosquito Larvicidal Activity

This study compared the larvicidal activity of the local plant, *A. arborescens*, and the marine sponge, *H. erectus*, derived extracts and fractions against the third larval instar of *Cx. pipiens* larvae mosquito at 24 and 48 h post-treatment.

As shown in Table 1, the crude methanolic extract of *A. arborescens* and its three fractions (*n*-hexane, dichloromethane, and ethyl acetate) exhibited larvicidal efficacy against *Cx. pipiens* larvae. The n-hexane fraction exhibited the highest larvicidal activity against the mosquito larvae in comparison to the other plant treatments, causing mortality rates ranging from 8% to 86% at concentrations of 200 to 500 µg/mL. The crude methanolic extract and dichloromethane fraction were moderately effective against mosquito larvae, while the lowest activity was observed for the ethyl acetate fraction, which induced mortality rates ranging from 4% to 64% at concentrations between 1800 µg/mL and 2500 µg/mL.

A Probit analysis of the *A. arborescens* crude methanolic extract and its fractions’ potency produced the 24 h and 48 h LC_50_ and LC_90_ values, respectively, shown in Table 2.

Non-overlapping confidence intervals between plant treatments confirmed statistically significant differences in larval sensitivity. However, comparisons of LC_50_ values across exposure periods (24 vs. 48 h) for the individual treatment showed overlapping confidence intervals, indicating no significant time-dependent differences in larval susceptibility except for the n-hexane fraction, whose LC_50_ reduction from 346.74 µg/mL to 289.78 µg/mL reflected a significant increase in potency over time.

Table 3 presents the larvicidal activity of the *H. erectus* marine sponge crude methanolic extract and its fractions against mosquito larvae. The results revealed a significant contrast in efficacy between the crude extract and its derived fractions. The crude methanolic extract showed lower activity, inducing mortality rates varying from 20% to 92% at concentrations ranging from 200 µg/mL to 500 µg/mL. On the contrary, the chloroform fraction exhibited remarkable activity, with a mortality rate of 22% at a low concentration of 50 µg/mL, escalating to 96% at a higher concentration of 100 µg/mL within 24 h post-treatment. The *n*-hexane and n-butanol fractions demonstrated comparable toxicity levels to the chloroform fraction against the targeted mosquito larvae.

A Probit analysis of the *H. erectus* methanolic, n-hexane, chloroform, and n-butanol fraction potency produced the 24 h and 48 h LC_50_ and LC_90_ values, respectively, shown in Table 4.

The effectiveness of the methanolic extract of *H. erectus* marine sponge against mosquito larvae was significantly different from its three derived fractions. This was evident by the non-overlapping confidence intervals for the LC_50_ values after the exposure periods. Essentially, the *n*-hexane, chloroform, and n-butanol fractions demonstrated greater larvicidal potency compared to the crude methanolic extract by approximately 4.1, 4.5, and 3.9 times, respectively. On the other hand, there was no significant difference among the three fractions themselves in terms of their efficiency, as indicated by the overlapping confidence limits of the LC_50_ values. This suggests that these fractions had similar toxic effects on the targeted larvae mosquitoes. Additionally, the larvicidal effectiveness of the crude methanolic extract and chloroform fraction of *H. erectus* marine sponge is significantly influenced by the duration of exposure, unlike the n-butanol and *n*-hexane fractions, as indicated by the non-overlapping in confidence limits for the LC_50_ values at two different periods.

### 3.2. Biotoxicity Against Non-Target Organisms

**Figure 2:**
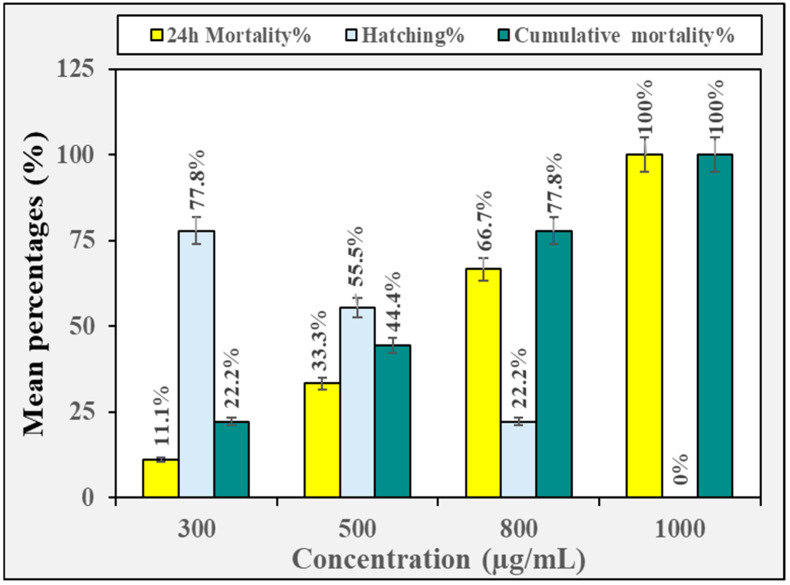
The toxicity effect of the crude methanolic extract of *H. erectus* marine sponge on the zebrafish embryos.

**Figure 3:**
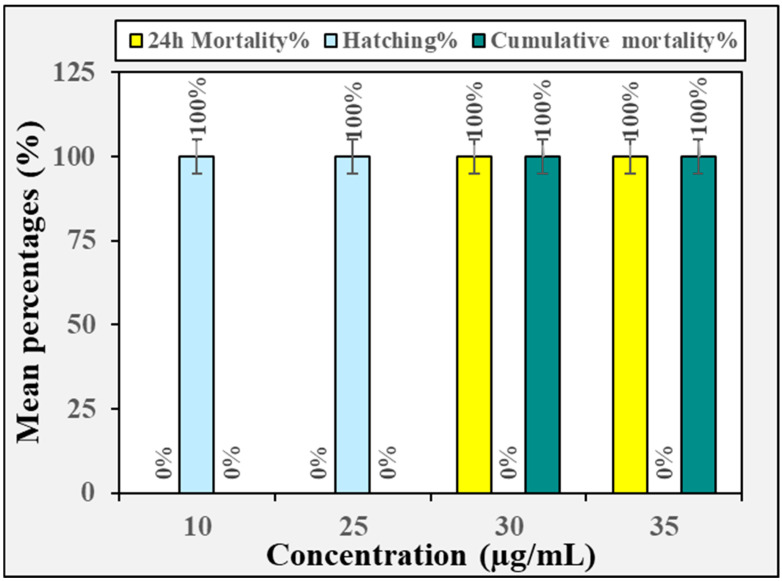
The toxicity effect of the *n*-hexane fraction derived from the crude methanolic extract of *H. erectus* marine sponge on the zebrafish embryos.

**Figure 4:**
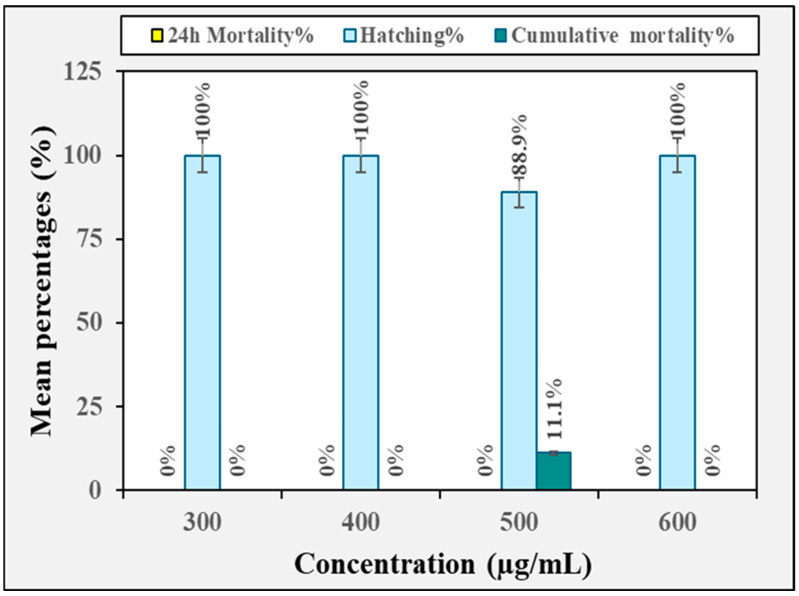
The toxicity effect of the chloroform fraction derived from the crude methanolic extract of *H. erectus* marine sponge on the zebrafish embryos.

**Figure 5:**
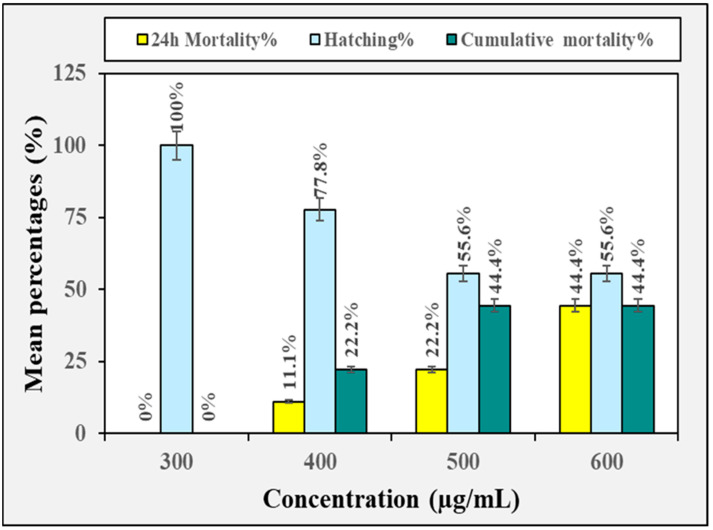
The toxicity effect of the *n*-butanol fraction derived from the crude methanolic extract of *H. erectus* marine sponge on the zebrafish embryos.

**Figure 6:**
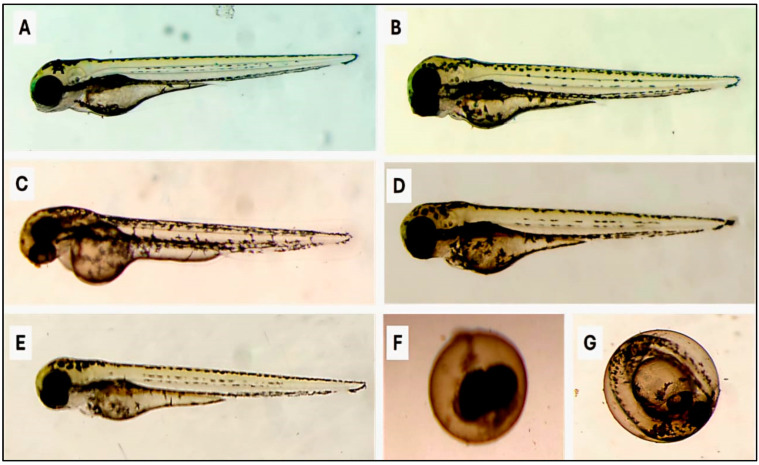
Biosafety evaluation of *H. eretus* in the zebrafish embryos. Representative micrographs of the zebrafish embryos at 3 days post-fertilization were treated with the crude methanolic extract of the marine sponge *H. erectus* and its various fractions. The embryos treated with the crude methanolic extract (**B**), as well as the *n*-hexane (<25 µL) (**C**), chloroform (**D**), and *n*-butanol (**E**) fractions, developed normally with no observable differences in morphology or growth compared to the control group (**A**). However, the embryos exposed to a higher concentration of *n*-hexane (>25 µL) showed complete lethality (**F**). Additionally, unhatched embryos were also observed in some treatments (**G**).

The toxicological effect of the crude methanolic extract of *H. erectus* and its fraction on zebrafish embryos is displayed in Figure 2, Figure 3, Figure 4, Figure 5 and Figure 6. The crude methanolic extract showed a concentration-dependent response of zebrafish embryo toxicity towards *H. erectus* crude and solvent fractions. There was a direct correlation between increasing extract concentrations and greater mortality percentages among the embryos, ranging from 22.2% at the lowest concentration to a complete mortality percentage of 100% at the highest concentration after 72 h of exposure (Figure 2). This increase in toxicity corresponded with reduced hatching percentages across all the tested concentrations, with the group exposed to 1000 µg/mL showing no successful hatching. The *n*-hexane fraction demonstrated a clear distinction in embryonic mortality and hatching success between lower and higher concentration groups (Figure 3). No mortality was observed at the lower concentrations of 10 and 25 µg/mL, and all the embryos hatched successfully. In contrast, a significant increase in mortality percentages was observed in the embryos exposed to higher extract concentrations, particularly at 30 and 35 µg/mL, where the mortality percentage reached 100%. Conversely, the survival of the zebrafish embryos was not adversely affected by the chloroform fraction across the tested concentration range of 300 to 600 µg/mL (Figure 4). Remarkably, all the chloroform fraction concentrations demonstrated a complete hatching rate, indicating that the extract does not pose a significant threat to embryo survival within this range. However, it is noteworthy that at the concentration of 500 µg/mL, a slight decrease in the hatching percentage was observed, with 88.9% of the embryos successfully hatching. The *n*-butanol fraction had variable impacts on zebrafish embryos depending on the concentration administered (Figure 5). No adverse effects were observed at 300 µg/mL, with a complete hatching percentage of the embryos. However, when the concentration was increased to 400 µg/mL, a notable mortality rate of 22.2% was recorded, indicating that this concentration begins to exert detrimental effects on embryo survival. Further increases in concentration to 500 µg/mL and 600 µg/mL resulted in an identical mortality percentage of 44.4%, suggesting a significant escalation in toxicity at these higher levels. The consistent mortality rate at both concentrations implies that the embryos experience similar levels of toxicological impact, regardless of the slight increase in concentration. Overall, the hatching embryos developed normally with no observable differences in morphology or growth compared to the control group (Figure 6).

The biosafety indexes (BIs) presented in Table 5 indicate that both the *n*-butanol fraction and crude methanolic extract of *H. erectus* are safe for the zebrafish embryos. The *n*-butanol fraction showed remarkable safety with a high BI of 8.85 and an LC_50_ of 616.47 µg/mL (551.40–854.78 µg/mL). The crude methanolic extract also demonstrated safety with a BI of 1.82 and an LC_50_ of 511 µg/mL (462.54–566.34 µg/mL). On the other hand, the determination of LC_50_ values for the *n*-hexane and chloroform fractions on the zebrafish embryos cannot be calculated due to insufficient data in the observed mortality percentages across the concentration ranges tested in this study. Consequently, this limitation prevented the reliable construction of a linearized concentration–response curve, which is essential for accurately calculating LC_50_ values. The significantly lower concentrations of *n*-hexane fraction showed toxicity in the zebrafish embryos as compared to its larvicidal activity in mosquitoes, strongly suggesting its high acute toxicity in the zebrafish embryos. Conversely, the chloroform fraction was safe even at significantly higher concentrations in the zebrafish embryos than those applied to mosquito larvae, suggesting the potential safety of this extract for the zebrafish embryos at the tested concentrations.

## 4. Discussion

The search for eco-friendly pesticide alternatives from natural sources, particularly for mosquito control, has gained significant attention, driven by a global shift toward eco-conscious solutions over harmful synthetic chemical insecticides. Nature harbors a wealth of bioactive compounds, with plants and marine organisms being vital sources of bioactive molecules, offering beneficial compounds for a wide range of applications, including mosquito control [24,37].

The study revealed significant variations in larvicidal efficacy among the tested extracts and fractions derived from both the plant and the marine sponge. Among the plant-derived extracts from *A. arborescens*, the *n*-hexane fraction was the most potent plant-based larvicide with LC_50_ of 346.74 μg/mL, showing significantly higher activity than the *A. arborescens* methanolic extract and other its fractions (dichloromethane and ethyl acetate).

The observed variability in larvicidal efficacy among *A. arborescens* crude extract and its fractions in this study can be attributed to the differing polarities of the solvents used in fractionation, which directly influence their ability to solubilize and concentrate bioactive constituents [38,39].

This finding supports the established mosquitocidal potential of various *Artemisia species*, known for their effectiveness against different mosquito developmental stages. The *A. arborescens n*-hexane fraction exhibited superior larvicidal activity, exceeding the effectiveness previously reported for extracts from French *Artemisia molinieri* (LC_50_ 9091 ppm) and *A. campestris var. glutinosa* (LC_50_ 9898 ppm) against *Cx. pipiens* larvae after 48 h [40]. Similarly, its efficacy surpassed that reported for Indian *A. absinthium* methanolic (LC_50_ 888.6 ppm) and ethanolic (LC_50_ 694.3 ppm) extracts against 4th instar *Aedes aegypti* larvae [41]. In contrast, essential oils derived from Moroccan *Artemisia species* displayed even stronger larvicidal activity against *Cx. pipiens*, outperforming the current study’s results with calculated LC_50_ values of 11.11 μg/mL for *A. arborescens*, 16.98 μg/mL for *A. absinthium*, and 19.07 μg/mL for *A. campestris* [42]. Likewise, Moroccan *A. flahaultii* (LC_50_ 29.328 ppm) and *A. annua* (LC_50_ 278.539 ppm) essential oils also demonstrated notable efficacy against the same species, while *A. aragonensis* (LC_50_ 2373.75 ppm) exhibited lower larvicidal activity than *the A. arborescens* methanolic extract and its fractions in this study, except for the ethyl acetate fraction [40].

The marine sponge *H. erectus* extracts showed promising results regarding larvicidal activity. Overall, these sponge-derived extracts (including both crude and fractionated forms) exhibited significantly higher potency compared to all the tested plant-derived extracts. This observation underscores the potential of marine organisms as a rich source of unique bioactive compounds for mosquito control. Notably, three *H. erectus* fractions—*n*-hexane, chloroform, and *n*-butanol—demonstrated remarkable efficacy with LC_50_ values of 68.39 μg/mL, 63.03 μg/mL, and 71.23 μg/mL, respectively. These fractions showed 3.9 to 4.5 times greater larvicidal activity than the crude methanolic sponge extract. This substantial increase in potency highlights the enhanced effectiveness achieved through fractionation, particularly for marine sponge extracts.

According to the available research, there is no specific information about the mosquitocidal or larvicidal activity of the marine sponge *H. erectus* against mosquitoes. Therefore, this study represents the first investigation into the larvicidal properties of the crude methanolic extract of *H. erectus* and its fractions derived using solvents of varying polarities on *Culex* larvae. This is particularly significant given the limited literature on the larvicidal effects of this sponge species, necessitating comparisons with other marine-derived agents. The *H. erectus* fractions demonstrated the highest larvicidal activity, surpassing previously reported results for the Indian marine sponge *Cliona celata*, which was tested against *Anopheles stephensi*, *Ae. aegypti*, and *Cx. quinquefasciatus*, with LC_50_ values ranging from 80.61 to 364.71 ppm [24,43]. Although the methanolic extract of *H. erectus* was the least potent among its fractions (LC_50_ = 280.74 μg/mL), it still outperformed the hexane extract of *C. celata* against *Aedes aegypti* (LC_50_ = 364.71 ppm). In contrast, significantly stronger larvicidal effects were reported for other marine-derived agents. For example, the Red Sea marine sponge *Amphimedon chloros*, when combined with carbon nanotubes, achieved an LC_50_ of 15.569 ppm against *Ae. aegypti* [44]. Similarly, a biosurfactant produced by *Enterobacter cloacae* SJ2, isolated from the marine sponge *Clathria* sp., exhibited a 48 h LC_50_ of 26.49 mg/L against *Cx. quinquefasciatus* [45], both demonstrate greater potency than the *H.* ***erectus*** extracts evaluated in this study.

The present study also sought to provide insights into the toxicological effects of *H. erectus* crude methanolic extracts and its fractions using the embryos of zebrafish as the vertebrate model. The zebrafish embryos have emerged as valuable models for assessing toxicity, offering several advantages over traditional mammalian models. Their rapid development, with major organs forming within 24 h post-fertilization, allows for the quick assessment of developmental toxicity [46,47]. Embryo transparency enables the real-time, non-invasive observation of morphological changes and organ development [48]. The zebrafish embryo toxicity test (ZFET) has shown a strong correlation with mammalian toxicity data, making it suitable for chemical screening and hazard assessment. This model is particularly useful for assessing specific organ toxicities, including neurotoxic, cardiotoxic, hepatotoxic, and genotoxic effects. Additionally, it offers ethical and cost-effective advantages, potentially reducing the use of mammalian models in early-stage toxicity testing [49].

The recent findings from the toxicology study revealed that the zebrafish embryos exhibited varying levels of response to the tested marine sponge over 72 h of exposure. They were least responsive to *n*-butanol fraction, with biosafety indexes of 8.85. Similarly, the zebrafish embryos showed less susceptibility to the crude methanolic extract, with a biosafety index of 1.82. In contrast, the *n*-hexane fraction demonstrated high toxicity levels at concentrations exceeding 25 µg/mL. Whereas, the chloroform fraction showed no toxic effects at most concentrations tested, except for slight toxicity at 500 µg/mL. These findings align with previous studies that have explored the toxicological impacts of marine sponge extracts on zebrafish embryos. For instance, A study by Carnovali et al. [50] revealed that eight compounds isolated from the marine sponge *Aplysina aerophoba* exhibited varying toxicity profiles in zebrafish embryos. While compounds showed mild toxicity at later stages, aerophobin-1 demonstrated low toxicity above 1 μM, and aerophobin-2 and LL-PAA216 were non-toxic. In contrast, uranidine and fistularin-3 exhibited high toxicity, with uranidine causing embryo death at all concentrations and fistularin-3 inducing death at 48 h post-fertilization and causing pericardial edema at higher concentrations. Hanif et al. reported that araguspongines alkaloid isolated from the marine sponge *Neopetrosia chaliniformis* was toxic to zebrafish embryos, with an LC_50_ value of 4.3 µg/mL at 48 hpf and 3.6 µg/mL at 72 hpf. The study also observed significant teratogenic effects, including malformations and cell death in embryos, with 76% exhibiting abnormalities [51]. The varying toxicity profiles observed across different fractions and concentrations in this study emphasize the need for further research to understand the underlying mechanisms of these differential toxicities and their potential implications for pharmaceutical and environmental applications.

## 5. Conclusions

This study is considered an initial step towards implementing natural products in the battle against this Filaria vector as it highlighted the promising potential of eco-friendly alternatives for mosquito control derived from natural sources. The significant variations in larvicidal efficacy observed among the tested extracts, with *A. arborescens* and the marine sponge *H. erectus* showing notable effectiveness, underline the richness of natural biodiversity in providing viable solutions to combat mosquito populations. Furthermore, the investigation into the toxicological effects utilizing zebrafish embryos provides critical insights into the safety profiles of these extracts. The varying responses observed across different fractions and concentrations highlight the need for careful assessment to balance efficacy against potential toxicity. Given the escalating concerns over synthetic insecticides and their environmental impact, the findings from this study advocate for further exploration and development of these natural extracts. Continued research could lead to the formulation of effective and safe mosquito control agents, contributing to sustainable pest management practices while minimizing ecological disruption. Overall, this study paves the way for the integration of natural products as viable alternatives in integrated pest management systems, promoting a healthier, more sustainable environment. This necessitates conducting more future studies for the phytochemical characterization of these extracts and their possible synergistic activity against mosquito larvae. Moreover, semi-field experiments should be conducted prior to a wide-range field application.

## Figures and Tables

**Table 1:** Larvicidal activity of the crude methanolic extract of *A. arborescens* and its fractions of various solvents against 3rd instar larvae of *Cx. pipiens* mosquito.

Extract/Fraction	Concentration (µg/mL)	Mean Mortality% ± SE	
		Exposure Period	
		24 h	48 h
Crude methanolic extract	1200	10 ± 3.16	14 ± 4.00
	1500	40 ± 6.32	52 ± 8.00
	1700	70 ± 4.47	76 ± 5.10
	2000	86 ± 5.10	94 ± 2.48
*n*-Hexane fraction	200	8 ± 4.90	16 ± 4.00
	300	32 ± 8.00	50 ± 8.60
	400	64 ± 7.48	84 ± 8.12
	500	86 ± 6.78	96 ± 4.00
Dichloromethane fraction	1200	10 ± 0.0	14 ± 3.16
	1500	28 ± 7.35	34 ± 8.12
	1700	40 ± 8.37	52 ± 10.7
	2000	78 ± 5.83	80 ± 10.5
Ethyl acetate fraction	1800	4 ± 2.45	6 ± 2.45
	2000	20 ± 5.48	20 ± 5.48
	2300	36 ± 8.72	44 ± 5.10
	2500	62 ± 12.0	70 ± 8.94

Each value is represented as the mean ± standard error (SE) of five replicates (*n* = 5), and each replicate had 10 individual larvae. No mortality was observed in the control groups.

**Table 2:** Probit analysis of the mortality percentages of the 3rd instar larvae of *Cx. pipiens* mosquito exposed to *A. arborescens* crude methanolic extract and its fractions of various solvents.

Exposure Period	Extract/Fraction	LC_50_ (µg/mL) (LCL-UCL)	LC_90_ (µg/mL) (LCL-UCL)	Slope ± SE	Chi-Square
24 h	Crude methanolic extract	1564.44 (1517.63–1611.56)	2046.76 (1951.60–2183.55)	10.981 ± 1.0	1.4786
	*n*-Hexane fraction	346.74 (328.86–365.45)	554.26 (509.29–621.47)	6.291 ± 0.567	0.927
	Dichloromethane fraction	1722.37 (1663.77–1791.66)	2377.80 (2212.36–2644.81)	8.634 ± 0.925	1.612
	Ethyl acetate fraction	2392.88 (2328.44–2478.21)	3006.58 (2832.64–3292.91)	12.926 ± 1.46	3.173
48 h	Crude methanolic extract	1491.22 (1442.63–1537.73)	1957.59 (1866.92–2089.13)	10.844 ± 1.04	1.169
	*n*-Hexane fraction	289.78 (274.33–304.49)	454.78 (426.62–492.57)	6.548 ± 0.507	0.928
	Dichloromethane fraction	1643.06 (1585.26–1706.68)	2312.53 (2153.37–2568.49)	8.634 ± 0.925	1.612
	Ethyl acetate fraction	2318.78 (2264.67–2384.08)	2862.38 (2726.98–3071.54)	14.012 ± 1.44	1.074

LC_50_—lethal concentration that kills 50% of the exposed larvae. LC_90_—lethal concentration that kills 90% of the exposed larvae. LCL represents the lower confidence limit, and UCL represents the upper confidence limit based on a 95% confidence interval. Values of different treatments with non-overlapping confidence limits are considered significantly different (*p* ≤ 0.5) according to Litchfield and Wilcoxon [36].

**Table 3:** Larvicidal activity of the crude methanolic extract of **H. erectus* marine sponge* and its fractions of various solvents against 3rd instar larvae of *Cx. pipiens* mosquito.

Extract/Fraction	Concentration (µg/mL)	Mean Mortality% ± SE	
		Exposure Period	
		24 h	48 h
Crude methanolic extract	200	20 ± 3.16	44 ± 3.16
	300	56 ± 5.10	66 ± 5.10
	400	82 ± 6.63	86 ± 5.10
	500	92 ± 5.83	98 ± 4.00
*n*-Hexane fraction	60	20 ± 3.16	26 ± 6.78
	70	64 ± 5.10	72 ± 9.17
	80	78 ± 3.74	84 ± 9.27
	100	94 ± 6.00	98 ± 2.00
Chloroform fraction	50	22 ± 7.35	30 ± 6.32
	70	68 ± 10.20	68 ± 5.83
	90	80 ± 8.94	82 ± 4.90
	100	96 ± 2.45	96 ± 4.00
*n*-Butanol fraction	60	32 ± 4.90	36 ± 6.00
	80	68 ± 4.90	72 ± 3.74
	120	80 ± 4.47	86 ± 5.10
	150	96 ± 2.45	98 ± 2.00

Each value is represented as a mean ± standard error (SE) of five replicates (*n* = 5), and each replicate had 10 individual larvae. No mortality was observed in the control groups.

**Table 4:** Probit analysis of the mortality percentages of the 3rd instar larvae of *Cx. pipiens* mosquito exposed to *H. erectus* marine sponge crude methanolic extract and its fractions of various solvents.

Exposure Period	Extract/Fraction	LC_50_ (µg/mL) (LCL-UCL)	LC_90_ (µg/mL) (LCL-UCL)	Slope ± SE	Chi-Square
24 h	Crude methanolic extract	280.74 (254.72–304.62)	470.44 (421.28–555.59)	5.717 ± 0.743	0.049
	*n*-Hexane fraction	68.39 (64.995–71.37)	89.65 (84.28–98.99)	10.903 ± 1.551	3.885
	Chloroform fraction	63.03 (58.00–67.38)	94.80 (87.18–107.28)	7.231 ± 0.939	3.482
	*n*-Butanol fraction	71.23 (62.03–78.78)	132.8 (117.3–161.1)	4.738 ± 0.680	3.418
48 h	Crude methanolic extract	225.98 (190.8–252.8)	426.01 (377.88–513.68)	4.654 ± 0.708	1.956
	*n*-Hexane fraction	65.54 (61.65–68.62)	86.79 (81.61–96.07)	10.509 ± 1.60	1.571
	Chloroform fraction	60.58 (55.55–64.78)	90.93 (84.34–101.04)	7.265 ± 0.893	2.368
	*n*-Butanol fraction	67.49 (58.85–74.43)	118.37 (105.43–142.02)	5.252 ± 0.785	2.810

LC_50_—lethal concentration that kills 50% of the exposed larvae. LC_90_—lethal concentration that kills 90% of the exposed larvae. LC_90_ represents the lower confidence limit, and UCL represents the upper confidence limit based on a 95% confidence interval. Values of different treatments with non-overlapping confidence limits are considered significantly different (*p* ≤ 0.5) according to Litchfield and Wilcoxon [36].

**Table 5:** Biosafety index of *H. erectus* marine sponge and its fractions against zebrafish embryos and median lethal concentration (LC_50_) values based on Probit analysis.

Extract/Fraction	LC_50_ (µg/mL) (UCL-LCL) *	Biosafety Index
Crude methanolic extract	511 (462.54–566.34)	1.82
*n*-Hexane fraction	not determined	not determined
Chloroform fraction	not determined	not determined
*n*-Butanol fraction	616.47 (551.40–854.78)	8.85

* LC_50_—lethal concentration that kills 50% of the exposed embryos. LC_90_ represents the lower confidence limit, and UCL represents the upper confidence limit based on a 95% confidence interval.

## Data Availability

The data presented in this study are included in the article. Further inquiries can be directed to the corresponding author.
